# Hierarchical Micro‐Nanoclusters of Bimetallic Layered Hydroxide Polyhedrons as Advanced Sulfur Reservoir for High‐Performance Lithium–Sulfur Batteries

**DOI:** 10.1002/advs.202003400

**Published:** 2021-01-29

**Authors:** Weilong Qiu, Gaoran Li, Dan Luo, Yongguang Zhang, Yan Zhao, Guofu Zhou, Lingling Shui, Xin Wang, Zhongwei Chen

**Affiliations:** ^1^ School of Materials Science and Engineering Hebei University of Technology Tianjin 300130 China; ^2^ School of Information and Optoelectronic Science and Engineering International Academy of Optoelectronics at Zhaoqing South China Normal University Guangdong 510006 China; ^3^ Department of Chemical Engineering University of Waterloo Waterloo ON N2L 3G1 Canada; ^4^ College of Materials Science and Engineering Nanjing University of Science and Technology Nanjing 210094 China; ^5^ South China Academy of Advanced Optoelectronics South China Normal University Guangdong 510006 China

**Keywords:** hollow polyhedrons, layered double hydroxides, lithium–sulfur batteries, nano‐micro hierarchies, spray drying

## Abstract

Rational construction of sulfur electrodes is essential in pursuit of practically viable lithium–sulfur (Li–S) batteries. Herein, bimetallic NiCo‐layered double hydroxide (NiCo‐LDH) with a unique hierarchical micro‐nano architecture is developed as an advanced sulfur reservoir for Li–S batteries. Compared with the monometallic Co‐layered double hydroxide (Co‐LDH) counterpart, the bimetallic configuration realizes much enriched, miniaturized, and vertically aligned LDH nanosheets assembled in hollow polyhedral nanoarchitecture, which geometrically benefits the interface exposure for host–guest interactions. Beyond that, the introduction of secondary metal intensifies the chemical interactions between layered double hydroxide (LDH) and sulfur species, which implements strong sulfur immobilization and catalyzation for rapid and durable sulfur electrochemistry. Furthermore, the favorable NiCo‐LDH is architecturally upgraded into closely packed micro‐nano clusters with facilitated long‐range electron/ion conduction and robust structural integrity. Due to these attributes, the corresponding Li–S cells realize excellent cyclability over 800 cycles with a minimum capacity fading of 0.04% per cycle and good rate capability up to 2 C. Moreover, highly reversible areal capacity of 4.3 mAh cm^−2^ can be achieved under a raised sulfur loading of 5.5 mg cm^−2^. This work provides not only an effective architectural design but also a deepened understanding on bimetallic LDH sulfur reservoir for high‐performance Li–S batteries.

## Graphical abstract

A unique NiCo‐LDH@rGO micro‐nano hierarchical composite is developed as a synergistic sulfur immobilizer and promoter to realize high‐efficiency lithium–sulfur battery chemistry. The unique architecture of NiCo‐LDH@rGO and its strong chemical interactions with the intermediate polysulfides afford large space for sulfur, enable expedite ion/mass transfer, and effectively restrain and catalyze the conversion of polysulfide.

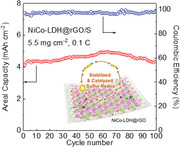

## 1 Introduction

Lithium–sulfur (Li–S) batteries play a critical role in the development of next‐generation energy storage systems, owing to their multiple advantages such as high theoretical energy density (2600 Wh kg^−1^), low cost, and environmental benignity.^[^ ^1^ ^]^ However, several intractable challenges are still plaguing this promising technology from yielding cogent practicability, which involves the insulating nature of Li_2_S and S in both electrical and ionic respects, the large volume variation during charge/discharge processes, and the “shuttle effect” regarding the dissolution and migration behaviors of the intermediate polysulfides (LiPS) upon battery operation.^[^ ^2^ ^]^ These disadvantages give rise to low sulfur utilization, poor cycling stability and rate capability, and serious Coulombic inefficiency, hindering the practical implementation of Li–S batteries.

During the past few decades, massive research efforts have been devoted to addressing these undesirable issues. Advanced sulfur reservoir is considered as a crucial strategy to improve the sulfur electrochemistry towards enhanced battery performance. Carbon materials, such as graphene,^[^ ^3^ ^]^ carbon nanotubes,^[^ ^4^ ^]^ porous carbons,^[^ ^5^ ^]^ etc., have been extensively studied due to their good conductivity, high porosity, and certain physical confinement to enhance the sulfur utilization and cycling stability. Albeit the considerable progress, the lack of chemical affinity between nonpolar carbons and weak‐polar LiPS is still struggling to bring forth sufficient sulfur immobilization and shuttle inhibition. Given this, polar materials such as heteroatom‐doped carbons,^[^ ^6^ ^]^ transition metal (TM)‐based inorganics,^[^ ^7^ ^]^ metal–organic frameworks (MOFs),^[^ ^8^ ^]^ etc. have been proposed and explored to impose additional chemical confinement towards further improvement of Li–S performance. Apart from sulfur confinements, the catalyzation of sulfur reactions is another critical aspect, and has been attracting enormous research attentions in the development of advanced Li–S batteries. Representatively, certain TMs/phosphides,^[^ ^9^ ^]^ metallic nanostructures, etc.^[^ ^10^ ^]^ have been demonstrated catalytically active to sulfur redox reactions that improves the overall battery reaction kinetics.

Recently, TM layered double hydroxide (TM‐LDH) has been emerging as a particularly promising sulfur reservoir in Li–S batteries. LDH is a class of ionic solids featuring the unique 2D layered structure, large surface area, and decent porosity, which is highly favorable to electrochemical energy storage and conversion systems.^[^ ^11^ ^]^ As for Li–S batteries, the LDH materials deliver several particular advantages: 1) the partial isomorphous substitution by high‐valence metal contributes to the positively charged surface,^[^ ^12^ ^]^ which could electrostatically trap the polysulfide anions; 2) the atomic‐level 2D structure determines a high proportion of surface atoms that renders an highly efficient utilization of active sites for host–guest interactions; and 3) the abundant hydroxyl groups on LDH surface are highly lithiophilic, which could serve as adsorptive sites towards LiPS. In view of these favorable features, LDH‐based sulfur electrodes have been exploited with certain progress.^[^ ^13^ ^]^ However, the critical roles of the bimetallic configuration as well as their mechanistic impacts on sulfur electrochemistry are still obscure, while rational architectural designs that guarantee electron/mass supplies and reliable structural robustness are still lacking and urgently calling for further and serious advances.

Herein, we developed a unique micro‐nano hierarchical construction of bimetallic NiCo‐LDH (NiCo‐LDH) as advanced sulfur reservoir for Li–S batteries. Compared with monometallic Co‐LDH (denoted as Co‐LDH), the introduction of secondary metal (Ni) regulates the hydrolysis and nucleation behaviors upon the chemical etching and reprecipitation processes, contributing to the more abundant, miniaturized, and vertically aligned NiCo‐LDH nanosheets that enrich the interface exposure for host–guest interactions. Beyond that, the bimetallic configuration endows the LDH surface chemistry with superior sulfur immobilization and catalyzation in comparison with the monometallic counterpart. On this basis, the obtained NiCo‐LDH was further architecturally upgraded via spray drying technique. The product delivers a sophisticated micro‐nano hierarchy with NiCo‐LDH polyhedrons tightly clustered and sufficiently wrapped by reduced graphene oxide thin shells (NiCo‐LDH@rGO), which favors the overall long‐range conductivity and structural integrity. Attributed to these architectural and chemical benefits, sulfur electrodes based on NiCo‐LDH@rGO realize outstanding cyclability over 800 cycles and superb rate capability up to 2 C, as well as high areal capacity and cyclic stability under a high sulfur loading.

## 2 Results and Discussion

**Scheme 1:**
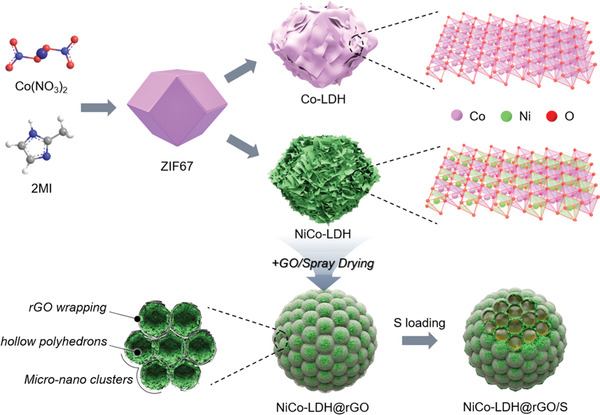
Scheme of the synthesis of Co‐LDH, NiCo‐LDH, NiCo‐LDH@rGO, and NiCo‐LDH@rGO/S composites.

**Figure 1:**
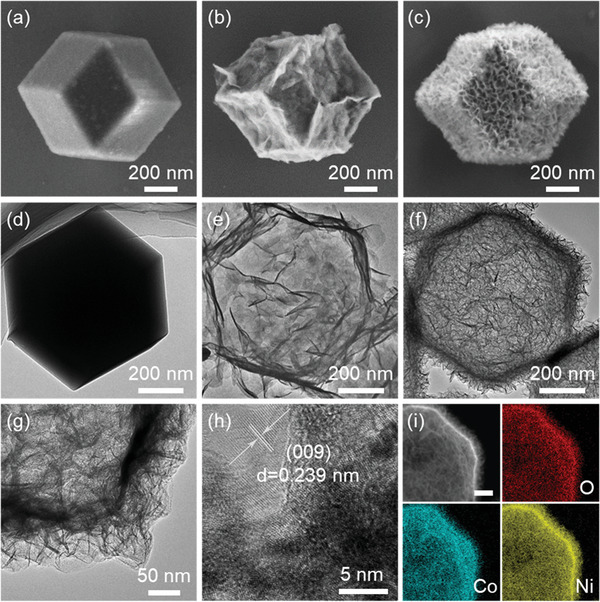
SEM images of a) ZIF67, b) Co‐LDH, and c) NiCo‐LDH. TEM images of d) ZIF67, e) Co‐LDH, and f,g) NiCo‐LDH. h) HRTEM image and i) elemental mapping (scale bar equals 100 nm) of NiCo‐LDH.

**Scheme** **1** illustrates the preparations of the monometallic Co‐LDH and bimetallic NiCo‐LDH, as well as the hierarchical NiCo‐LDH@rGO and the according sulfur‐based composite. First, zeolitic imidazolate framework‐67 (ZIF67) nanocrystals were prepared through the conventional solution‐based method,^[^ ^14^ ^]^ which delivers a typical dodecahedral morphology with uniform size and smooth surface as depicted in **Figure** **1**a and Figure S1a, Supporting Information. After that, the Co‐ and Ni‐etchings were performed to convert the ZIF67 particles into polyhedral assemblies of Co‐LDH and NiCo‐LDH nanosheets, respectively. As for Co‐LDH, the ZIF67 was etched by the protons generated through the hydrolysis of Co^2+^, while Co^2+^ ions were partially oxidized by NO_3_^−^ ions and dissolved oxygen in the solution. Since Co^2+^ holds a stronger affinity to the Co‐containing template, the deposition of Co^2+^ along the templates is relatively easy even with fewer nucleation sites, which contributes to sparser nanosheets with large planar size (Figure 1b; Figure S1b, Supporting Information). On the contrary, nickel nitrate undergoes more drastic hydrolysis due to its lower pH value in ethanol. Hence, more nucleation sites are generated, resulting in densified and miniaturized LDH nanosheets along the polyhedral shells (Figure 1c; Figure S1c, Supporting Information).^[^ ^15^ ^]^ Apparently, compared with Co‐LDH, the bimetallic NiCo‐LDH with denser nanosheets is capable of affording more functional surfaces for sulfur redox reactions as well as the host–guest interactions.

Further morphological and microstructural details can be gained from transmission electron microscopy (TEM) observation. The ZIF67 presents the typical solid polyhedral particle as shown in Figure 1d. In comparison, distinct hollow structure can be observed for both Co‐LDH (Figure 1e) and NiCo‐LDH (Figure 1f,g) with the shells constructed by ultrathin LDH nanosheets, which accords with the SEM results and further verifies the successful conversion from ZIF67 to hydroxides via the chemical etching. Particularly, it can be noticed that NiCo‐LDH is endowed with significantly smaller, denser, and compactly assembled nanosheets along the polyhedral shells compared with that in Co‐LDH. This is determined by the chemical affinity between etching agent and template as well as the pH value of the etching environment, which regulates the hydrolysis and nucleation reactions as discussed above. Moreover, legible lattice fringe can be obtained in the high‐resolution TEM (HRTEM) image of NiCo‐LDH (Figure 1h; Figure S2, Supporting Information), which shows an interplanar spacing of 0.239 nm in correspondence to the (009) plane of NiCo‐LDH, suggesting a good crystallinity.^[^ ^16^ ^]^ In addition, the elemental mapping in Figure 1i confirms the uniform distributions of Ni, Co, and O in the as‐prepared NiCo‐LDH.

**Figure 2:**
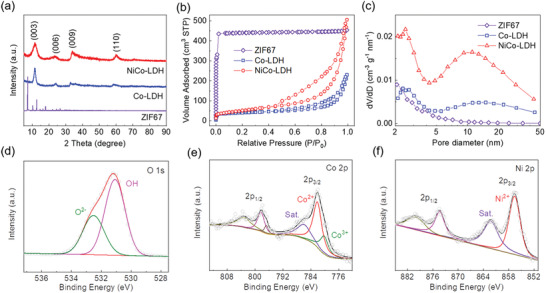
a) XRD patterns, b) N_2_ adsorption–desorption isotherms, and c) pore size distributions of ZIF67, Co‐LDH, and NiCo‐LDH. XPS d) O 1s, e) Co 2p, and f) Ni 2p spectra of NiCo‐LDH.

The compositional features of the products were probed by X‐ray diffraction (XRD) measurement. As shown in **Figure** **2**a, ZIF67 shows typically a group of sharp peaks, while the Ni‐etching and Co‐etching give rise to the broadened peaks locating at around 11.5°, 23.37°, 33.9°, and 60.0°, referring to the (003), (006), (009), and (110) facets, respectively, in good consistent with those in literatures.^[^ ^17^ ^]^ This compositional variation confirms the successful preparation of LDH materials, which can be also reflected by the color change among the samples in Figure S3, Supporting Information. The parent ZIF67 shows a typical purple color, which turns into light green and light rose color after the Ni‐etching and Co‐etching, respectively. The porous textures of different samples were compared by N_2_ adsorption–desorption measurement. As shown in Figure 2b and Figure S4, Supporting Information, ZIF67 exhibits a typical type‐I isotherm curve and strong pore size distribution at around 0.8 nm, indicating its dominating microporous feature. By contrast, the formation of Co‐LDH and NiCo‐LDH transforms the isotherm into type IV with a significant enhancement in mesoporosity and macroporosity. The specific surface area is ≈1786.29, 152.7, and 186.79 m^2^ g^−1^ for ZIF67, Co‐LDH, and NiCo‐LDH, respectively, based on Brunauer–Emmett–Teller (BET) theory. The evolution of porous texture can be further perceived from the pore size distributions based on BJH model as presented in Figure 2c. Notably, the conversion from ZIF67 into Co‐LDH and NiCo‐LDH mitigates the microporous dominance with significantly enriched mesoporosity in the product matrix. Meanwhile, this effect is more significant in the NiCo‐LDH scenario compared with that of Co‐LDH, which is capable of providing large spaces for sulfur accommodation, abundant pathways for ion/mass transfer, and sufficient exposure of active interfaces for sulfur electrochemical reactions.

X‐ray photoelectron spectroscopy (XPS) analysis was performed to study the surface chemistry of NiCo‐LDH. The survey spectrum in Figure S5, Supporting Information, confirms the elemental composition by showing sharp characteristic peaks of Ni, Co, and O. Meanwhile, the strong peak assigned to the —OH species in the O 1s spectrum confirms hydroxide dominance in the as‐prepared NiCo‐LDH (Figure 2d).^[^ ^18^ ^]^ Moreover, the Co 2p spectrum exhibits two sets of peaks attributed to the Co 2p_3/2_ and Co 2p_1/2_ sublevels as shown in Figure 2e. The Co 2p_3/2_ band can be differentiated into three subpeaks at 780.3, 782.2, and 786.2 eV ascribed to the Co^3+^, Co^2+^, and the satellite peak, respectively, suggesting a partial oxidization of Co. The Ni 2p XPS spectrum also shows the Ni 2p_3/2_ and 2p_1/2_ levels, while Ni^2+^ and satellite peak can be fitted at 856.6 and 862.3 eV, respectively (Figure 2f).^[^ ^19^ ^]^ These results well consist with the literatures and confirm the well‐defined NiCo‐based hydroxide structure.

**Figure 3:**
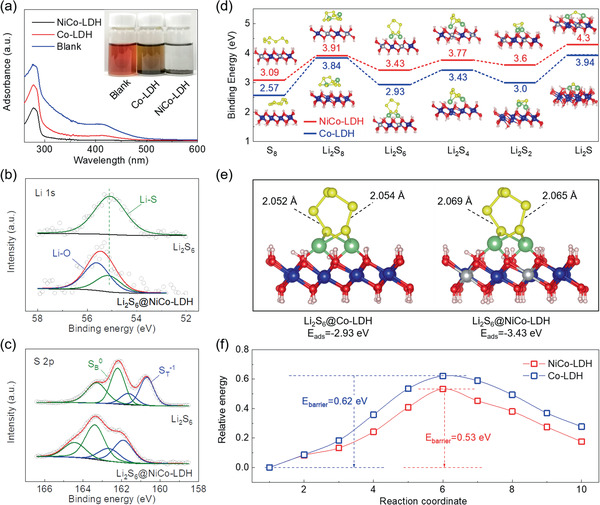
a) UV–vis spectra and optical images (inset) of LiPS solutions adsorbed by ZIF67, Co‐LDH, and NiCo‐LDH. b) Li 1s and c) S 2p spectra of Li_2_S_6_ before and after adsorbed on NiCo‐LDH. d) Adsorption energies of LiPSs on Co‐LDH and NiCo‐LDH surfaces. e) Geometrically stable configuration of Li_2_S_6_ adsorption on Co‐LDH and NiCo‐LDH. f) Energy barriers upon the Li_2_S decomposition on Co‐LDH and NiCo‐LDH surfaces.

Given this, the interactive chemistry between LiPS and different LDHs was further compared. A direct visual adsorption test was first conducted as shown in **Figure** **3**a (inset). The 5 × 10^−3^ m Li_2_S_6_/(1,2‐dimethoxyethane (DME)+1,3‐dioxolane (DOL)) solution was immersed with different absorbents and rested for 8 h. By comparison, the LiPS solution turns into almost colorless and transparent after adsorbed by NiCo‐LDH, while considerable yellowish color can be still observed for that with Co‐LDH, intuitively manifesting the stronger LiPS adsorption by the bimetallic NiCo‐LDH. This result can be also confirmed by the UV–vis spectra in Figure 3a. The UV–vis profile of blank Li_2_S_6_ solution shows two absorbance peaks at 277 and 410 nm, referring to the S_6_^2−^ and S_4_^2−^ species, respectively.^[^ ^20^ ^]^ It is noted that these peaks undergo more drastic intensity reduction after immersed with NiCo‐LDH, which strongly indicates the less LiPS residue in the supernatant and the higher LiPS adsorbability of NiCo‐LDH compared with its bimetallic Co‐LDH counterpart. In view of this, the underlying adsorption mechanism was probed by XPS analysis. Compared with the sole Li–S peak in the Li 1s spectrum of bare Li_2_S_6_, the Li_2_S_6_@NiCo‐LDH composite shows an additional strong peak at 55.6 eV assigned to the Li–O bonding (Figure 3b), suggesting the formation of a “lithium bond”‐like interaction. Moreover, the S 2p spectrum of bare Li_2_S_6_ shows typically two pairs of peaks at 160.7 and 162.2 eV assigned to the terminal S (S_T_^−1^) and bridging S (S_B_^0^), respectively (Figure 3c).^[^ ^21^ ^]^ It can be perceived that both these peaks experience a considerable shift to a higher binding energy range after adsorption by NiCo‐LDH, manifesting the decrease of electron cloud density in S atoms attributed to the chemical interactions with Li_2_S_6_ and NiCo‐LDH.

Beyond that, the LiPS–LDH interactions were further explored by the density functional theory (DFT) calculations. Figure 3d compares the adsorption energies of a series of sulfur species on Co‐LDH and NiCo‐LDH surface, accompanied by the accordingly geometrically stable adsorption configurations. It is worth noting that the NiCo‐LDH delivers higher chemical affinity to all range of LiPS with constantly higher adsorption energies compared with those for Co‐LDH, which strongly verifies its superior sulfur adsorbability in consistent with the visual adsorption test above. Representatively, the Li_2_S_6_ adsorptions on Co‐LDH and NiCo‐LDH surfaces are depicted in Figure 3e. It can be perceived that Li atoms tend to bond with oxygen to form the “lithium bond” that bridges the NiCo‐LDH or Co‐LDH with LiPS, which echoes well with the XPS result. The corresponding charge difference density patterns are presented in Figure S6, Supporting Information. As expected, O and Li serve as electron donors and acceptors, respectively, to establish the “S–Li–O” bridged bonding configuration. Notably, the Li_2_S_6_@NiCo‐LDH experiences a more significant charge transfer upon such bonding compared with that for Li_2_S_6_@Co‐LDH, suggesting a stronger chemical interaction. As a result, the bimetallic configuration enables a higher adsorption energy of –3.43 eV than its monometallic counterpart (–2.93 eV), which confirms its favorably higher LiPS adsorbability and is expected to impose stronger sulfur immobilization against the shuttle effect. Apart from that, elongated S—S bonds can be noticed after adsorption by NiCo‐LDH compared with that by Co‐LDH, suggesting an easier bond breakage for sulfur redox reactions. In addition, the potential kinetic improvement was also investigated specifically focusing on the Li_2_S decomposition behavior, considering its particularly sluggish reaction kinetics in sulfur electrochemistry. As shown in Figure 3f and Figure S7, Supporting Information, the bimetallic NiCo‐LDH surface enables an energy barrier of 0.53 eV for Li_2_S decomposition, which is lower than that of the monometallic Co‐LDH (0.62 eV). This result strongly demonstrates the great capability of NiCo‐LDH in facilitating the sulfur redox reactions, which is promising to ensure a high‐efficiency sulfur electrochemistry with good rate capability.

**Figure 4:**
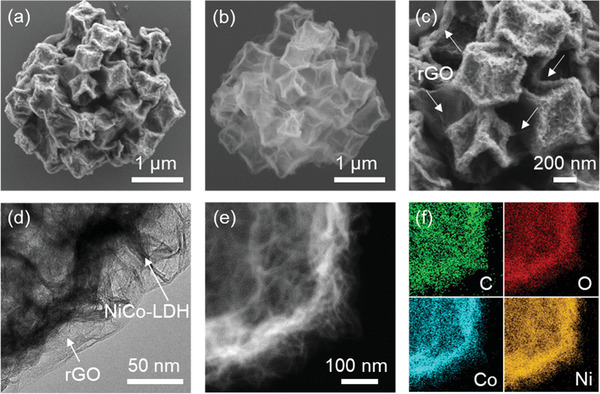
SEM images of NiCo‐LDH@rGO under a) low‐voltage of 2 kV and b) high‐voltage of 20 kV. c) High‐magnification SEM image, d) HRTEM image, and e,f) element mapping of NiCo‐LDH@rGO.

In view of these structural and chemical superiorities, the bimetallic NiCo‐LDH was further architecturally upgraded via spray drying technique, which is aimed to enhance the overall conductivity and structural integrity (Scheme 1). As shown in **Figure** **4** and Figure S8, Supporting Information, the obtained NiCo‐LDH@rGO composite shows a decent monodispersion with a fairly uniform size of around 3 µm. A closely packed hierarchical architecture can be observed for NiCo‐LDH@rGO, where the nanometric NiCo‐LDH polyhedrons were aggregated into a micrometric cluster (Figure 4a). The high‐voltage SEM image reveals the well‐maintained hollow character of the NiCo‐LDH units (Figure 4b). Meanwhile, the rGO thin films tightly wrap the polyhedron individuals as well as the overall clusters, affording an interconnected conductive framework and also a mechanical reinforcement (Figure 4c). The rGO thin layers can be clearly observed on the NiCo‐LDH surface as depicted the TEM image (Figure 4d). The successful rGO wrapping can be also confirmed by the color change of the sample (Figure S3, Supporting Information), as well as the emergence of a new broad peak at around 26.4° in the XRD pattern, which is assigned to the (002) plane of rGO (Figure S9, Supporting Information). The homogenous element distribution in NiCo‐LDH@rGO was also demonstrated by the element mapping as shown in Figure 4e,f. Apart from that, the porous feature of NiCo‐LDH@rGO was studied as presented in Figure S10, Supporting Information. After the rGO wrapping, the NiCo‐LDH@rGO maintains a similar isotherm profile to NiCo‐LDH, and a decent porosity with a specific surface area of 164.24 m^2^ g^−1^. The porous and hollow features are expected to offer large space for sulfur accommodation and fluent channels for ion/mass transfer. Meanwhile, the unique micro‐nano architectural hierarchy with tight rGO wrapping could also strengthen the long‐range electron/ion conduction among these nano‐subunits as well as increasing the structural integrity upon chemical/electrochemical process.

**Figure 5:**
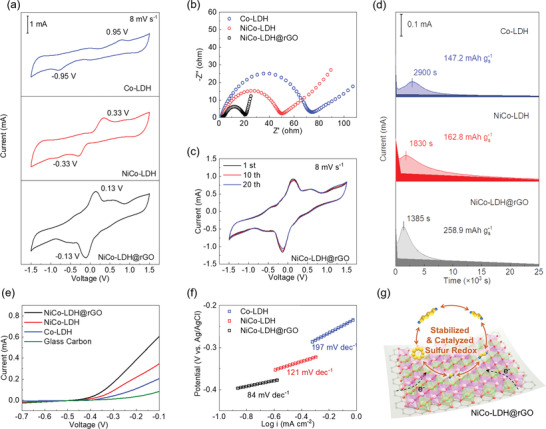
a) CV profiles and b) EIS spectra of symmetric cells based on different electrodes. c) CV profiles of NiCo‐LDH@rGO symmetric cell during 20 cycles at 8 mV s^−1^. d) Li_2_S deposition, e) Li_2_S oxidization profiles and f) the Tafel plots accordingly on different electrode surfaces. g) Schematic illustration of sulfur adsorption and catalyzation on NiCo‐LDH@rGO.

Based on the above results, sulfur redox behaviors on different host materials, i.e., Co‐LDH, NiCo‐LDH, and NiCo‐LDH@rGO, were compared first by symmetric‐cell measurements. The symmetric cells were fabricated using the identical electrodes and Li_2_S_6_‐contained electrolyte (see details in Experimental Section). The cyclic voltammetry (CV) profiles of symmetric cells clearly show the sharper peak shape, higher current response, and smaller redox potential gap (–0.13/0.13 V) for NiCo‐LDH@rGO cell compared with the Co‐LDH (–0.95/0.95 V) and NiCo‐LDH (–0.33/0.33 V) counterparts, indicating the faster LiPS conversions thereon (**Figure** **5**a). This kinetic enhancement can be also supported by the electrochemical impedance spectroscopy (EIS) results (Figure 5b), where the NiCo‐LDH@rGO cell exhibits the lowest charge‐transfer impedance among the different cells. Moreover, the well‐maintained CV profile upon 20 cycles signifies the good reversibility of LiPS conversions in NiCo‐LDH@rGO electrode (Figure 5c). Apart from that, the Li_2_S redox behaviors were also studied by Li_2_S precipitation and oxidization tests on different electrode surfaces. As shown in Figure 5d, the Li_2_S precipitation on NiCo‐LDH@rGO delivers the largest capacity of 258.9 mAh g^−1^ and earliest current peak at 1385 s with sharpest peak shape compared with those for NiCo‐LDH (162.8 mAh g^−1^, 1830 s) and Co‐LDH (147.2 mAh g^−1^, 2900 s) electrodes, indicating that NiCo‐LDH@rGO effectively facilitates the conversion of LiPS into insoluble Li_2_S. In addition, the Li_2_S oxidization was evaluated by linear sweep voltammetry (LSV) measurement, where the NiCo‐LDH@rGO electrode exhibits obviously the smallest onset potential as well as the highest current response among different electrodes at a scanning rate of 10 mV s^−1^ (Figure 5e). Accordingly, the Tafel plot shows the much lower slope of 84 mV dec^−1^ for NiCo‐LDH@rGO compared with those for NiCo‐LDH (121 mV dec^−1^) and Co‐LDH (197 mV dec^−1^), indicating its lowest energy barrier and fastest kinetics for Li_2_S decomposition, which is in good accordance with the DFT result (Figure 5f). The desirable kinetic improvement in NiCo‐LDH@rGO can be mainly attributed to several aspects: 1) the highly porous and hollow architecture provides sufficient space for electrolyte infiltration and uniform sulfur accommodation, as well as abundant interfaces for favorable host–guest interactions; 2) the strong chemical interactions between the bimetallic NiCo‐LDH and LiPS effectively confine the active species on the catalytic surface and lower their energy barrier for redox reactions; and 3) the architectural upgrade by spray drying renders compactly clustered NiCo‐LDH subunits with tight rGO wrapping, which further enhances the overall long‐range electron/ion conductions for fast electrochemical reactions (Figure 5g).

Besides, the kinetic improvement can be also reflected by the Li^+^ diffusion coefficient (*D*_Li_^+^) measured in coin‐cell configuration. Sulfur composites, i.e., Co‐LDH/S, NiCo‐LDH/S, and NiCo‐LDH@rGO/S, were prepared via conventional melt‐diffusion method (see details in Experimental Section). The successful sulfur loading was verified by XRD and N_2_ adsorption–desorption measurements (Figures S11 and S12, Supporting Information). The sulfur contents were determined ≈73, 71, and 70 wt% for NiCo‐LDH@rGO/S, NiCo‐LDH/S, and Co‐LDH/S, respectively, by thermogravimetric analysis (TGA) (Figure S13, Supporting Information). Figure S14a–c, Supporting Information, shows the CV curves of different electrodes at varied scanning rates. The correlation between peak current and the square root of scan rates (*υ* ^0.5^) at different anodic (A) and cathodic (B and C) peaks are presented in Figure S14d–f, Supporting Information. Compared with NiCo‐LDH/S and Co‐LDH/S, the cell based on NiCo‐LDH@rGO/S exhibits obviously higher slopes for all these peaks, demonstrating its highest *D*_Li_^+^ and the most facilitated Li^+^ diffusion among these samples, which further and consistently confirms the best sulfur reaction kinetics in NiCo‐LDH@rGO electrode.

**Figure 6:**
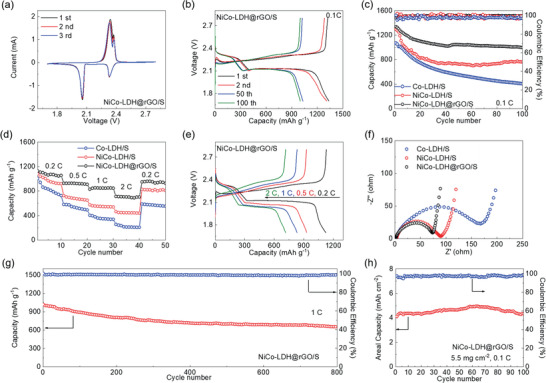
a) CV curves and b) charge–discharge profiles of NiCo‐LDH@rGO/S electrodes. c) Cycling and d) rate performance comparison between different electrodes. e) Voltage profiles of NiCo‐LDH@rGO/S electrode at varied current rates. f) EIS spectra of different electrodes. g) Long‐term cycling performance of NiCo‐LDH@rGO/S electrode at 1 C. h) Cycling behavior of NiCo‐LDH@rGO/S electrode at 0.1 C under sulfur loading of 5.5 mg cm^−2^.

Further electrochemical evaluations were performed in coin‐cell confirmation for different electrodes. **Figure** **6**a shows the CV profile of NiCo‐LDH@rGO/S electrode at initial several cycles. The cathodic scanning brings about two characteristic peaks at 2.33 and 2.06 V, referring to the sulfur reductions into high‐order soluble polysulfide (Li_2_S*_x_*, 4 ≤ *x* ≤ 8) and subsequently into low‐order insoluble Li_2_S_2_/Li_2_S, respectively. Meanwhile, anodic peaks at around 2.35 V can be attributed to their reverse conversions back into element sulfur.^[^ ^22^ ^]^ Notably, the CV curves well overlap upon the several cycles, demonstrating its good electrochemical reversibility.^[^ ^23^ ^]^ Meanwhile, it can be also noticed that the NiCo‐LDH@rGO/S electrode exhibits sharper peaks with narrower gaps for each pair of redox peaks compared with those for Co‐LDH/S and NiCo‐LDH/S electrodes (Figure S15, Supporting Information), suggesting the enhanced reaction kinetics by NiCo‐LDH@rGO. Accordingly, the galvanostatic charge/discharge profile of NiCo‐LDH@rGO/S electrode shows a two‐plateau discharge curve and one charging slope due to the multistep sulfur electrochemistry (Figure 6b). The voltage profile well maintains even after 100 cycles, further suggesting the stable sulfur reactions. Figure 6c compares the cycling performances of different electrodes. Impressively, the NiCo‐LDH@rGO/S electrode delivers a high initial capacity of 1336 mAh g^−1^ at 0.1 C, indicating a good sulfur utilization. Besides, an excellent cycling stability can be obtained with a capacity retention of 1000 mAh g^−1^ (74.8% of its initial value) after 100 cycles, which is much higher than those of Co‐LDH/S (406 mAh g^−1^, 37.9%) and NiCo‐LDH/S (763 mAh g^−1^, 58.9%) electrodes. A constantly higher Coulombic efficiency can be also achieved for NiCo‐LDH@rGO/S electrode in comparison with others. These results strongly indicate the effective shuttle inhibition and reaction stabilization attributed to the favorable sulfur immobilization by NiCo‐LDH@rGO as discussed above. Apart from that, the rate capabilities of different electrodes were also examined by multi‐rate cycling (Figure 6d). High capacities of 1129, 930, 831, and 713 mAh g^−1^ at 0.2 C, 0.5 C, 1 C, and 2 C, respectively, can be retained for NiCo‐LDH@rGO/S electrode, while the Co‐LDH/S and NiCo‐LDH/S electrodes suffer from fast capacity fading to 271 and 466 mAh g^−1^ at 2 C rate. This kinetic difference can be also perceived from the voltage profiles at varied current rates in Figure 6e and Figure S16, Supporting Information. NiCo‐LDH@rGO/S electrode is capable of retaining the two‐plateau discharge profile at raised rate up to 2 C, whereas the severe electrochemical polarization induces serious deformations of the voltage profiles for NiCo‐LDH/S and Co‐LDH/S electrodes. In addition, the EIS spectra also illustrate the lowest charge‐transfer impedance for NiCo‐LDH@rGO/S electrode, further illustrating its great superiority in facilitating sulfur redox reactions (Figure 6f). The superior rate performance originates from the advantageous sulfur adsorption and catalyzation accomplished by the chemical interactions between NiCo‐LDH and LiPS, while the unique micro‐nano hierarchical structure affords fast electron and ion supports.

Furthermore, the long‐term cyclability of NiCo‐LDH@rGO/S electrode was explored by galvanostatic cycling at 1 C. As shown in Figure 6g, NiCo‐LDH@rGO/S electrode remains a considerably high capacity of 658 mAh g^−1^ even after 800 cycles, corresponding to a minimum capacity fading rate of 0.04% per cycle. Meanwhile, a high Coulombic efficiency can be sustained, indicating the efficient and stable sulfur electrochemistry. Beyond that, NiCo‐LDH@rGO/S electrode with higher sulfur loading of 5.5 mg cm^−2^ was also prepared and evaluated to pursue higher energy density and practical viability. The result shows that the NiCo‐LDH@rGO/S electrode is still capable of yielding a decently high areal capacity of 4.3 mAh cm^−2^ after 100 cycles with a steady cycling behavior under the high‐loading configuration (Figure 6h). Besides, the as‐developed NiCo‐LDH@rGO/S electrode also delivers a highly competitive performance among the recently reported LDH‐based electrodes (Table S1, Supporting Information), further verifying its favorable structural benefits. These results collectively demonstrate the great superiorities of the unique micro‐nano hierarchical NiCo‐LDH@rGO design in promoting and stabilizing sulfur reactions for high‐performance Li–S batteries.

## 3 Conclusion

In summary, we have developed a unique NiCo‐LDH@rGO micro‐nano hierarchical composite as advanced sulfur reservoir for Li–S batteries. The bimetallic configuration realized a more enriched, miniaturized, and vertically aligned NiCo‐LDH hollow polyhedron compared with the monometallic Co‐LDH counterpart, which not only geometrically benefits sulfur accommodation, ion transfer, and active exposure, but also renders a favorable surface chemistry for strong sulfur immobilization and catalyzation. On this basis, an architectural upgrade was further implemented to construct the closely packed NiCo‐LDH@rGO clusters, where the unique micro‐nano hierarchy combined with the tight rGO wrapping ensures fast long‐range electron/ion conduction and robust structural integrity. As a result, a fast and durable sulfur electrochemistry can be achieved, which enables outstanding cyclability over 800 cycles and great rate capability up to 2 C, as well as decent performance under a high sulfur loading of 5.5 mg cm^−2^. This work offers a deepened understanding on LDH‐based sulfur electrochemistry as well as an advanced architectural design for high‐performance Li–S batteries, which could also enlighten the rational material engineering in other energy storage and conversion areas.

## 4 Experimental Section

##### Preparation of ZIF67

The ZIF67 was prepared as previously reported.^[^ ^24^ ^]^ Typically, 1.64 g 2‐methylimidazole (2MI) and 1.45 g Co(NO_3_)_2_·6H_2_O were dissolved in 125 mL methanol, respectively. Then, the 2MI solution was quickly added into Co(NO_3_)_2_·6H_2_O solution under vigorous stirring for 30 min. After that, the mixed solution was aged at 25 °C for 24 h. The solid product of ZIF67 was then filtered and washed by ethanol for three times followed by drying at 70 °C for 24 h.

##### Preparation of NiCo‐LDH and Co‐LDH

First, solution containing 0.365 g Ni(NO_3_)_2_·6H_2_O in 50 mL ethanol (solution A) and suspension containing 100 mg ZIF67 in 50 mL ethanol (suspension B) were prepared, respectively, and heated to 78 °C. The solution A was then slowly added into suspension B, and refluxed at 78 °C for 1 h.^[^ ^25^ ^]^ After cooling down naturally, the light green precipitate was collected, washed with ethanol, and dried overnight at 70 °C to obtain NiCo‐LDH. For comparison, the monometallic Co‐LDH was prepared through the same procedures but using 0.376 g Co(NO_3_)_2_·6H_2_O instead of Ni(NO_3_)_2_·6H_2_O as the etching agent.

##### Preparation of NiCo‐LDH@rGO

A total of 0.5 g NiCo‐LDH powder was added into GO solution (2 mg mL^−1^), which corresponds to a NiCo‐LDH to rGO mass ratio of 5:1, and subject to ultrasonic dispersion for 1 h. Thereafter, the suspension was spray dried at 200 °C under a flow rate of 3 mL min^−1^. The black product was collected and denoted as NiCo‐LDH@rGO.

##### Preparation of Sulfur‐Based Composites

The NiCo‐LDH@rGO/S composite was prepared through a typical melt‐impregnation method.^[^ ^26^ ^]^ The obtained NiCo‐LDH@rGO was mixed with element sulfur in weight ratio of 1:3, which was subsequently sealed in an autoclave and thermally treated at 155 °C for 12 h. The NiCo‐LDH@rGO/S was collected after cooling to ambient temperature. For comparison, the NiCo‐LDH/S and Co‐LDH/S composites were also obtained via the same process but with NiCo‐LDH and Co‐LDH as the sulfur host, respectively.

##### Material Characterizations

XRD patterns were recorded by Bruker D8 diffractometer equipped with Cu‐K*α* radiation source. The morphological details were obtained by field emission scanning electron microscopy (Sigma 500) combined with energy‐dispersive X‐ray spectroscopy and HRTEM (JEM‐2100F, JEOL). The N_2_ adsorption–desorption was conducted to study the pore characters by a V‐Sorb 2800P surface area and pore distribution analyzer instrument. The surface chemical states were analyzed by XPS (Thermo Fisher Scientific, ESCALAB 250Xi, USA). TGA (PerkinElmer, Series 7) was carried out to determine the sulfur weight contents at a heating rate of 5 °C min^−1^ under nitrogen atmosphere.

##### Symmetric Cell Characterization

The symmetric cells were prepared by using different samples (Co‐LDH, NiCo‐LDH, or NiCo‐LDH@rGO) as identical electrodes with 0.2 m Li_2_S_6_‐contained electrolyte in the CR2032 coin‐cell configuration.^[^ ^27^ ^]^ The CV profiles were collected at 8 and 12 mV s^−1^ for comparison and stability study, respectively, within a potential rage of –1.5 to 1.5 V. EIS spectra were recorded within the frequency range of 100 kHz to 0.1 Hz at an amplitude of 5 mV.

##### Li_2_S Precipitation and Oxidization

Coin cells were fabricated using NiCo‐LDH@rGO (or Co‐LDH, NiCo‐LDH) as cathode, Li foil as anode, and (0.25 m Li_2_S_8_+1 m lithium bis(trifluoromethane sulfonimide) (LiTFSI))/tetraglyme as catholyte for Li_2_S precipitation measurement.^[^ ^28^ ^]^ Cells were first galvanostatically discharged to 2.06 V to consume most of the high‐order LiPS, and then kept potentiostatic discharging at 2.05 V for Li_2_S nucleation and grown until the current dropped below 0.01 mA. The lighter color indicates the precipitation of Li_2_S, whereas the darker color represents the reduction of LiPS according to previous reports.^[^ ^29^ ^]^

The Li_2_S oxidization behaviors were investigated by LSV at a scanning rate of 10 mV s^−1^. A three‐electrode configuration was employed with platinum sheet as counter electrode, Ag/AgCl electrode as reference, and 0.1 m Li_2_S/methanol solution as electrolyte.^[^ ^30^ ^]^

##### Electrochemical Characterizations

The electrochemical evaluations were performed in CR2032‐type coin cells with NiCo‐LDH@rGO/S (or NiCo‐LDH/S, Co‐LDH/S) as cathode, lithium foil as anode, and Celgard 2400 membrane as separator. The sulfur electrodes were prepared by casting the slurry containing active materials (80 wt%), Super P (10 wt%), and polyvinylidene fluoride (10 wt%) in *N*‐methyl pyrrolidinone solvent onto Al foil followed by vacuum drying at 60 °C for 12 h. The electrolyte contained 1 m LiTFSI in a mixed solvent of DME and DOL (1:1 in volume) with 1 wt% lithium nitrate (LiNO_3_) as additive. The sulfur loading for regular electrodes was 2 mg cm^−2^, while higher sulfur loading of 5.5 mg cm^−2^ was employed for high‐loading configuration. The electrolyte‐to‐sulfur ratio (E/S) for regular loading electrodes was 15 mL g^−1^, which was decreased to 6.5 mL g^−1^ for high‐loading configurations. The galvanostatic charging and discharging were conducted by Neware battery tester within the voltage range of 1.7 to 2.8 V (vs Li/Li^+^, hereafter inclusive). The cyclic CV and EIS measurements were carried out by Princeton electrochemical workstation (Versa STAT4). CV profiles at varied scanning rate from 0.1 to 0.5 mV s^−1^. The *D*_Li_^+^ is correlated to the fitted slope based on the Randles–Sevcik diffusion equation^[^ ^31^ ^]^

(1) $$I_{P}=\left(2.69\;\times 10^{5}\right)\;n^{1.5}aD_{\mathrm{Li}}^{0.5}v^{0.5}C_{\mathrm{Li}}$$

where *I*_p_ corresponds to the peak current, *n* refers to the number of electrons transferred in the redox event, *a* is the active area of the electrode, *D* presents the Li^+^ diffusion coefficient, *υ* is the scan rate, and *C*_Li_ refers to the Li^+^ concentration in electrolyte.

##### Computational Characterizations

The DFT calculations were carried out using the Vienna ab initio simulation package program with Perdew–Burke–Ernzerhof for the exchange and correlation energy terms. A series of LiPS including Li_2_S, Li_2_S_2_, Li_2_S_4_, Li_2_S_6_, and Li_2_S_8_ were employed to study their adsorption behaviors on NiCo‐LDH surface. The cut‐off energy was set as 400 eV, and a 2 × 2 × 1 Monkhorst−Pack k‐point sampling was used. A vacuum height of 15 Å was used between successive slabs. Structures were fully relaxed and the maximum ionic force was 0.03 eV Å^−1^. The bonding energies of LiPS and S_8_ with NiCo‐LDH were calculated as follows

(2) $$E_{\mathrm{ads}}=E_{\mathrm{LiPS}/\mathrm{host}}-E_{\mathrm{LiPS}}-E_{\mathrm{host}}$$

where *E*_LiPS/host_ represents the total energy of the adsorption system, and *E*_LiPS_ and *E*_host_ are the energy of the isolated LiPS and host (i.e., Co‐LDH or NiCo‐LDH), respectively.

## Conflict of Interest

The authors declare no conflict of interest.

## Supporting information

Supporting Information
