# Supplementary material for: Hierarchical Micro‐Nanoclusters of Bimetallic Layered Hydroxide Polyhedrons as Advanced Sulfur Reservoir for High‐Performance Lithium–Sulfur Batteries
Source: Adv Sci (Weinh). 2021 Jan 29;8(7):2003400. doi: 10.1002/advs.202003400 (PMC8025003; doi:10.1002/advs.202003400)
Supplement: Supplementary file 1 — Supporting Information [file ADVS-8-2003400-s001.pdf]

© 2021 Wiley-VCH GmbH

ADVANCED SCIENCE
Open Access

Supporting Information

for *Adv. Sci.,* DOI: 10.1002/advs.202003400

Hierarchical Micro-nanoclusters of Bimetallic Layered Hydroxide Polyhedrons as Advanced Sulfur Reservoir for High-Performance Lithium-Sulfur Batteries

Weilong Qiu, Gaoran Li, Dan Luo, Yongguang Zhang, Yan Zhao*, Guofu Zhou, Lingling Shui, Xin Wang, and Zhongwei Chen**

Supporting Information

Hierarchical Micro-nanoclusters of Bimetallic Layered Hydroxide Polyhedrons as Advanced Sulfur Reservoir for High-Performance Lithium-Sulfur Batteries

*Weilong Qiu, Gaoran Li, Dan Luo, Yongguang Zhang**, *Yan Zhao*, Guofu Zhou, Lingling Shui, Xin Wang, and Zhongwei Chen**

W. Qiu, Prof. Y. Zhang, Y. Zhao

School of Materials Science and Engineering, Hebei University of Technology, Tianjin 300130, China

Email: yongguangzhang@hebut.edu.cn (Y. Zhang); yanzhao1984@hebut.edu.cn (Y. Zhao)

W. Qiu, Dr. X. Wang, Dr. Y. Zhang, Prof. L. Shui

School of Information and Optoelectronic Science and Engineering & International Academy of Optoelectronics at Zhaoqing, South China Normal University, Guangdong 510006, China

Prof.G. Zhou, Dr.X. Wang

South China Academy of Advanced Optoelectronics, South China Normal University, Guangdong 510006, China

Dr. G. Li

College of Materials Science and Engineering, Nanjing University of Science and Technology, Nanjing 210094, China

Prof. Z. Chen, Dr. G. Li, Dr. D. Luo

Department of Chemical Engineering, University of Waterloo, Waterloo, ON N2L 3G1, Canada

Email: zhwchen@uwaterloo.ca

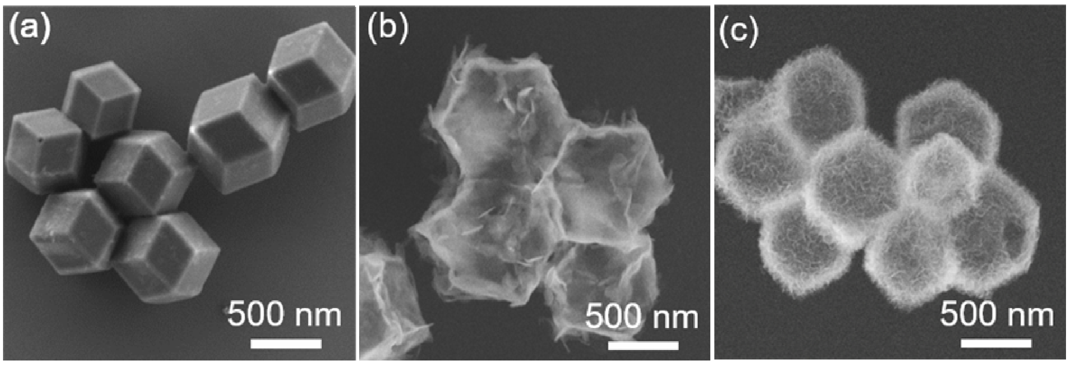

Figure S1. SEM images of (a) ZIF67, (b) Co-LDH, and (c) NiCo-LDH under hig-voltage of 10 kV.

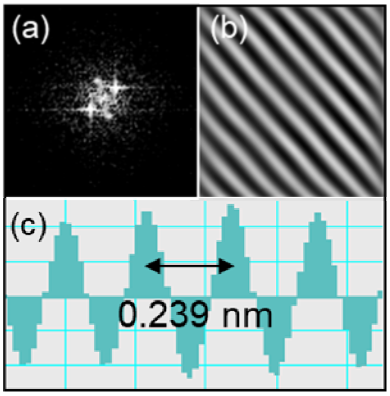

Figure S2.(a) FFT pattern, (b)inverse FFT images and (c) lattice spacing profiles at the selected area of NiCo-LDH.

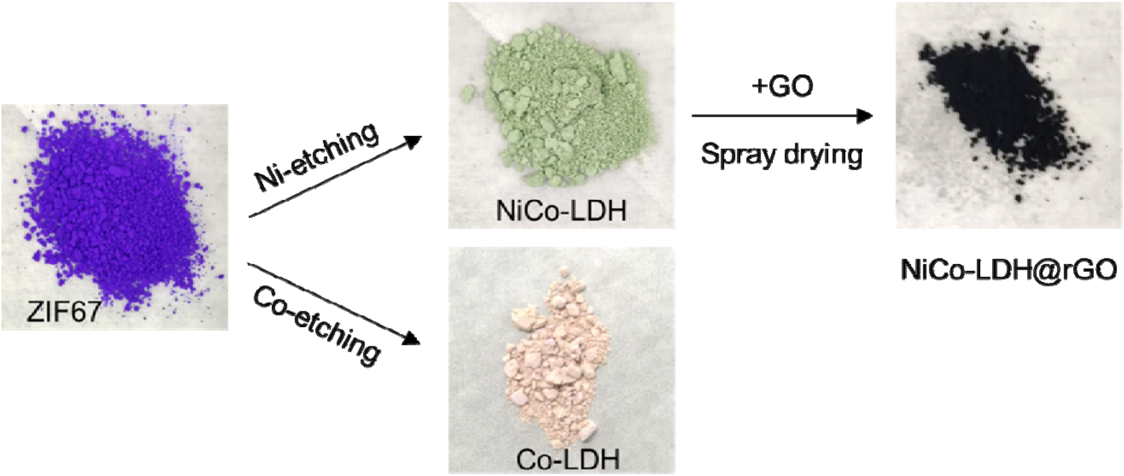

Figure S3. Color changes upon the preparation of ZIF67, Co-LDH, NiCo-LDH, and NiCo-LDH@rGO.

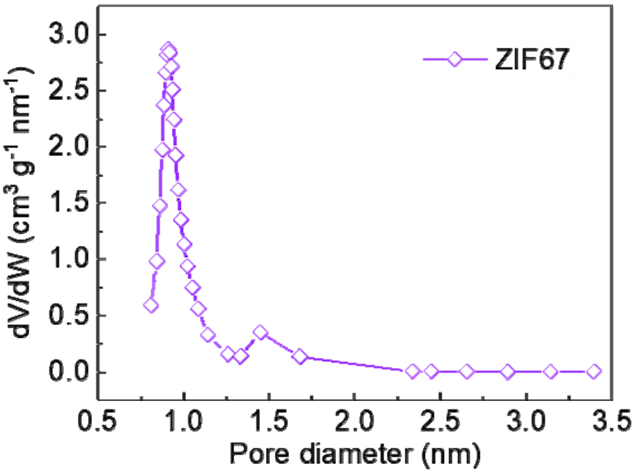

Figure S4. Pore size distribution based on H-K model confirming the micropore dominance in ZIF67.

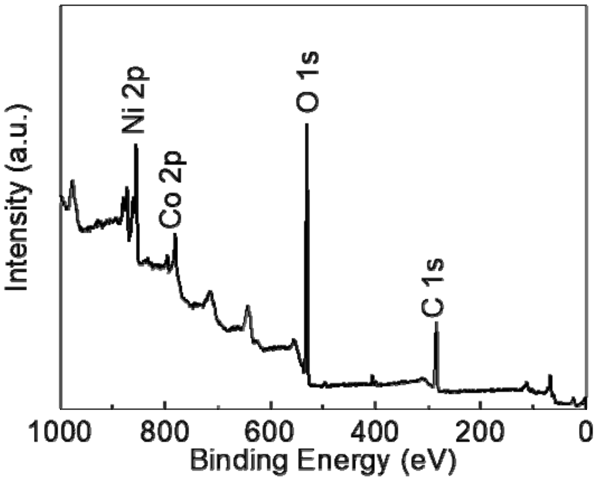

Figure S5. XPS survey spectra of NiCo-LDH.

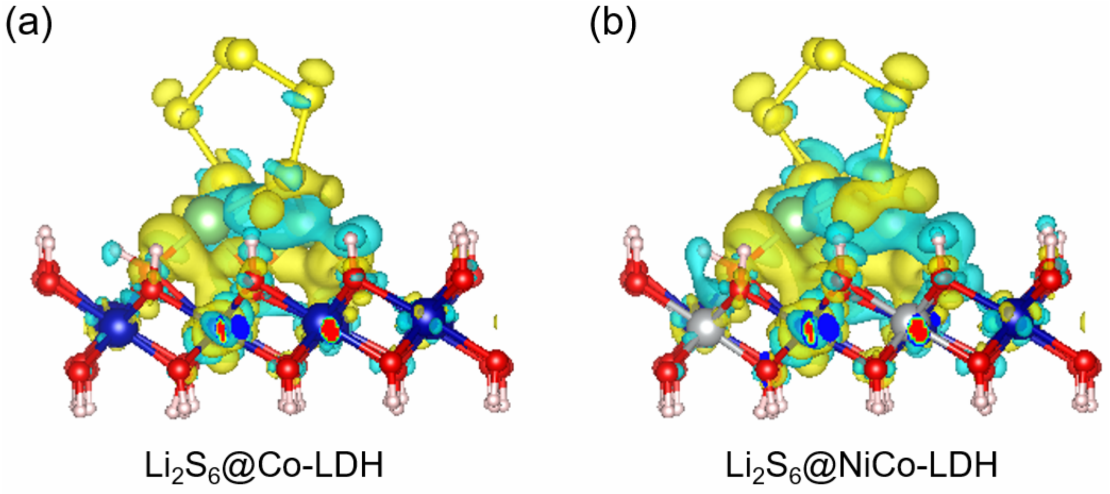

Figure S6. Charge density difference plots of Li_2S_6 adsorption on (a) Co-LDH and (b) NiCo-LDH surafecs, where the yellow and cyan isosurfaces present charge accumulation and depletion, respectively.

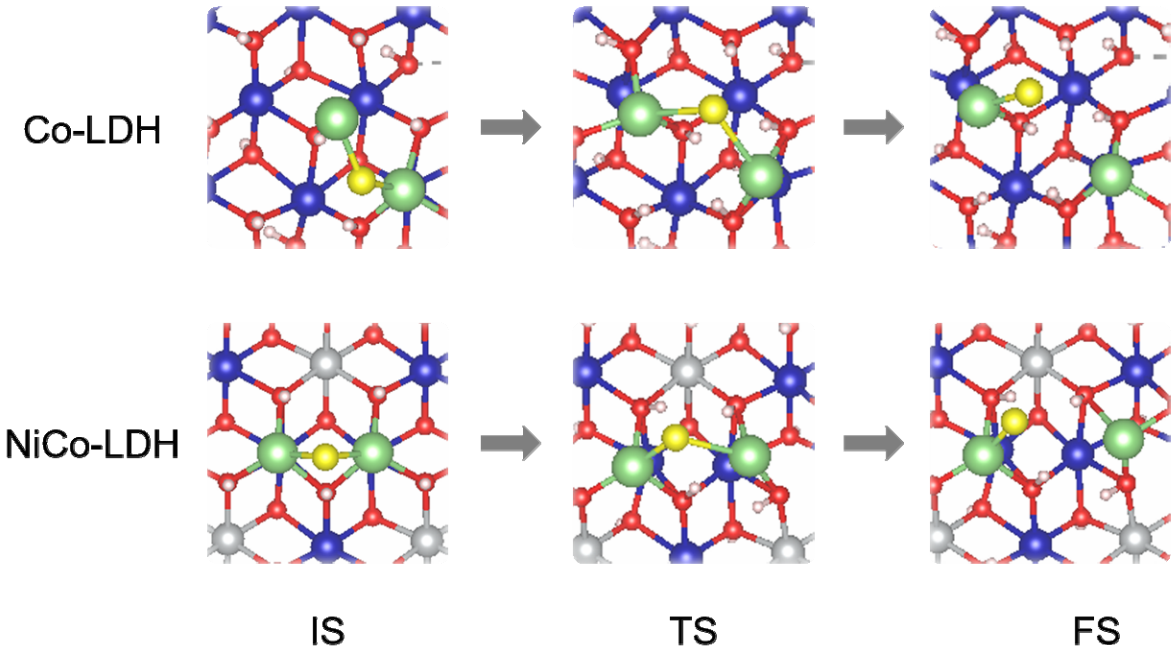

Figure S7. Configuration variations upon the Li_2S decomposition on Co-LDH and NiCo-LDH surfaces (IS, TS, and FS stand for initial, transitional, and final state, respectively)

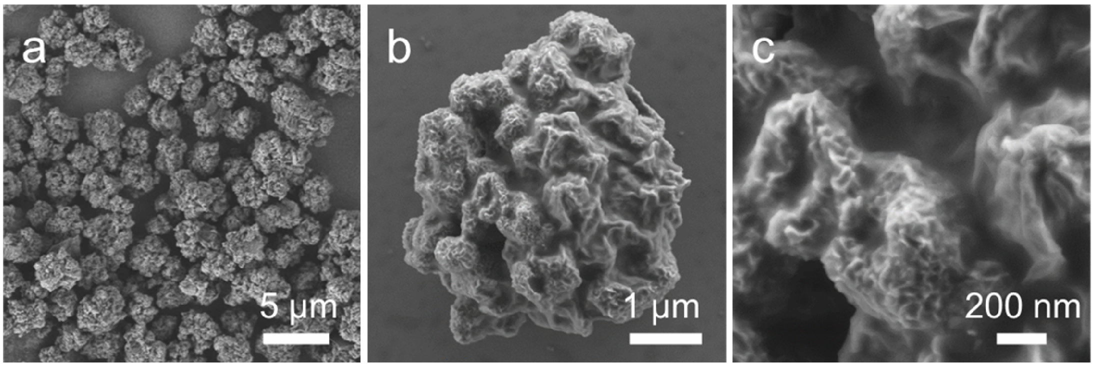

Figure S8. SEM images of NiCo-LDH@rGO under differnet magnifications.

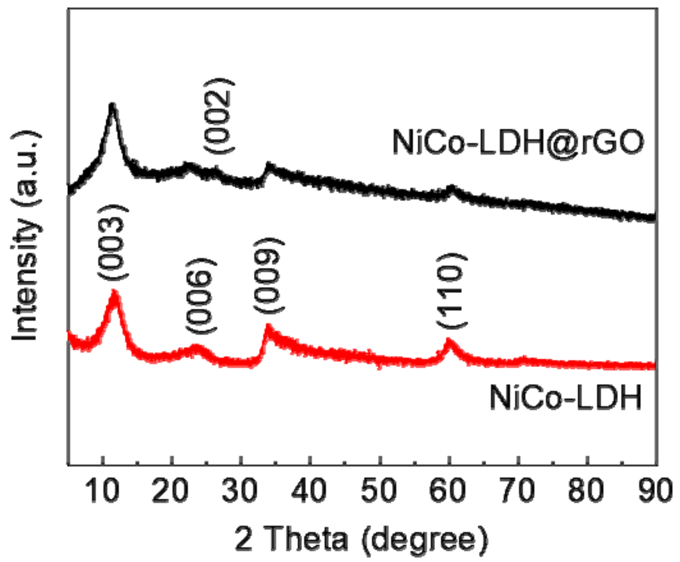

Figure S9. XRD patterns of NiCo-LDH@rGOand NiCo-LDH.

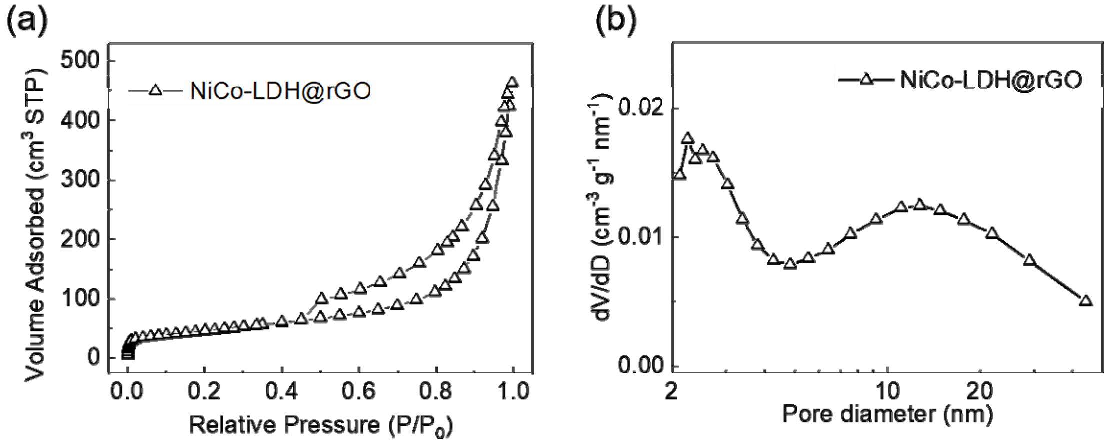

Figure S10. (a) N_2 adsorption-desorption isotherms, and (b) BJH pore size distribution of NiCo-LDH@rGO.

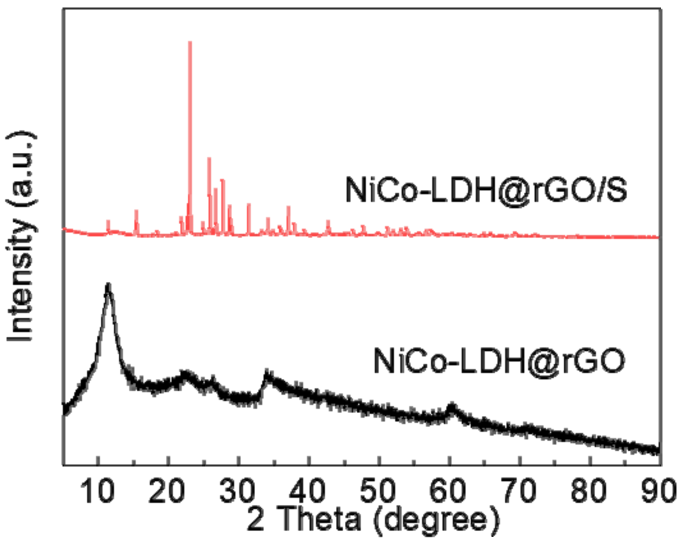

Figure S11. XRD patterns of NiCo-LDH@rGO before and after sulfur loading.

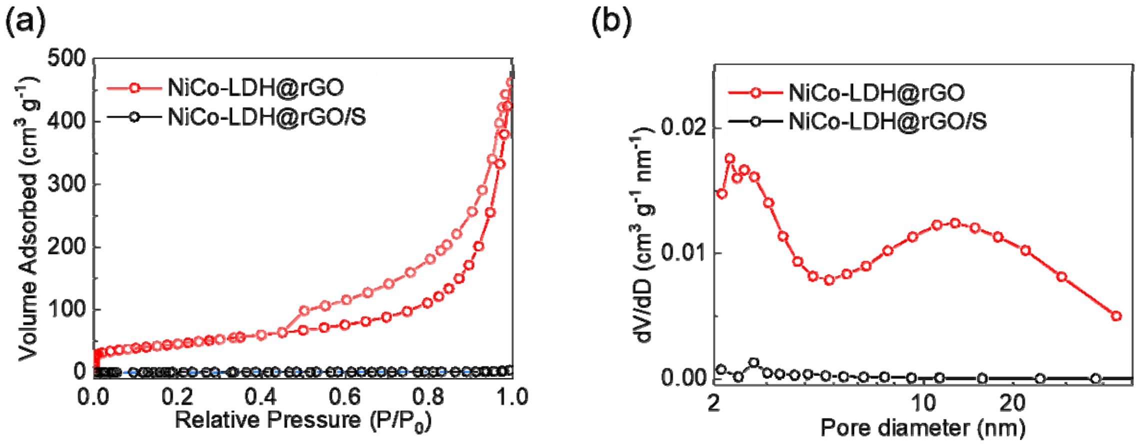

Figure S12. (a) N_2 adsorption-desorption isotherms, and (b) BJH pore size distribution of NiCo-LDH@rGO and NiCo-LDH@rGO/S.

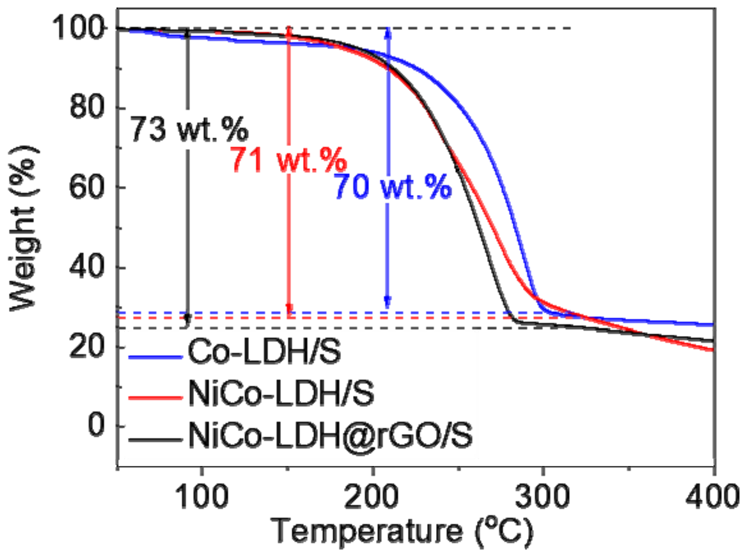

Figure S13. TGA curves of Co-LDH/S, NiCo-LDH/S, and NiCo-LDH@rGO/S composites.

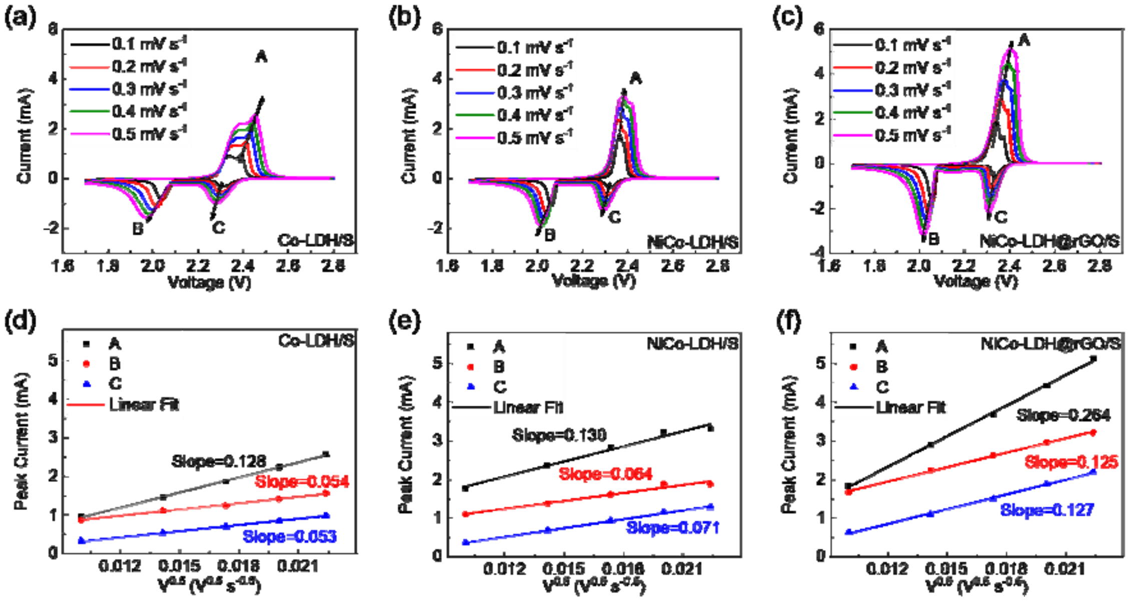

Figure S14. (a-c) CV curves at varied scanning rates and (d-f) the corresponding linear fittings between I_p and $\upsilon^{1/2}$ for (a, d) Co-LDH/S, (b, e) NiCo-LDH/S, and (c, f) NiCo-LDH@rGO/S electrodes.

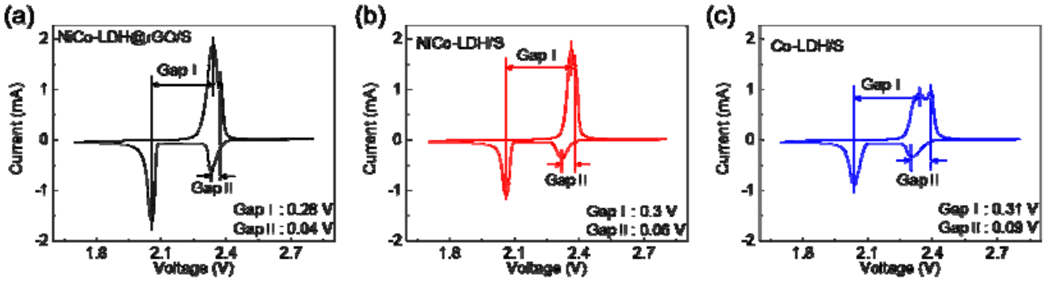

Figure S15. Typical CV curves of cells with (a) NiCo-LDH@rGO/S, (b) NiCo-LDH/S and (c) Co-LDH/S electrodes.

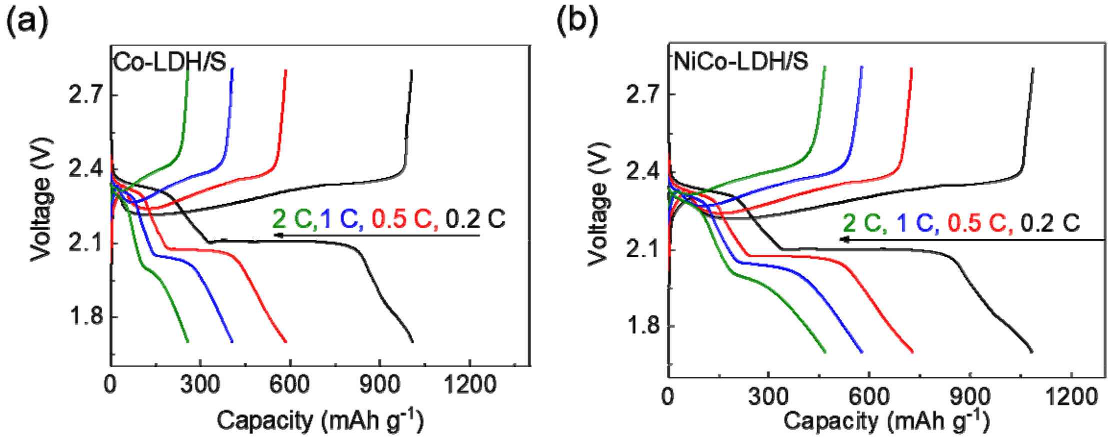

Figure S16. Voltage profiles of (a) Co-LDH/S and (b) NiCo-LDH/S electrodes at varied current rates.

Table S1. Performance comparison among different LDH-based sulfur electrodes.

Sample	Initial capacity (mAh g^{-1})/rate	Capacity retention (mAh g^{-1}) /rate/cycle	Rate capacity (mAh g^{-1})/rate	Areal capacity (mAh cm^{-2})/cycle/ S loading (mg cm^{-2})	Ref.
S/LDH/rGO	958/0.2C	394/1C/200	502/2C	-	[1]
S@Ni/Fe-LDH	1091/0.2C	501/1C/1000	633/2C	-	[2]
LDH/S/rGO	1380/0.2C	566/2C/1000	707/2C	3.7/200/4.2	[3]
CH@LDH/S	1014/0.1C	491/0.5C/100	500/1C	-	[4]
NiAl@PAB/S	792/0.5C	473/1C/300	614/3C	-	[5]
**NiCo-LDH@rGO/S**	**1336/0.1C**	**658/1C/ 800**	**713/2C**	**4.3/100/5.5**	**This work**

[1] F. C. Xu, C. W. Dong, B. Jin, H. Li, Z. Wen, Q. Zhang, *J. Electroanal. Chem.***2020**, 876, 114545.

[2] J. T. Zhang，Z. Li, Y. Chen, S. Y. Gao, X. W. Lou, *Angew. Chem., Int. Ed.* **2018**, 57, 10944.

[3] S. T. Liu, X. Y. Zhang, S. T. Wu, X. Chen, X. J. Yang, W. B. Yue, J. Lu, W. Z. Zhou, *ACS Nano***2020**, 14, 8220.

[4] J. T. Zhang, H. Hu, Z. Li, X. W. Lou, *Angew. Chem., Int. Ed.* **2016,**55, 3982.

[5] S. X. Chen, Z. L. Wu, J. H. Luo, X. X. Han, J. Wang, Q. Deng, Z. L. Zeng, S. G. Deng, *Electrochim. Acta***2019**, 312, 109.
